# The Protracted Civil and Armed Conflicts in Ethiopia Fueling the COVID-19-Related Health Crisis: Perspective on Building a Resilient Health System to Shocks

**DOI:** 10.4314/ejhs.v33i5.17

**Published:** 2023-09

**Authors:** Yifru Berhan, Beyeberu Assefa, Awoke Tassew, Wondosen Mengiste, Alegnta Gebreyesus, Zelalem Geletu, Ketema Muluneh, Getachew Asfaw, Samuel Abera, Abebe Negesso, Samuel Abebe, Habtamu Abelneh, Ermias Deju, Tsedeke Mathewos

**Affiliations:** 1 St Paul's Hospital Millennium Medical College; 2 UNFPA-Ethiopia; 3 Ministry of Health-Ethiopia; 4 Individual consultant

**Keywords:** Armed conflict, civil conflict, Ethiopia, health infrastructure damage, health system disruption, humanitarian-development nexus, internally displaced persons

## Abstract

Prior to the intensified civil and armed conflicts in Ethiopia, remarkable progress was made in the health sector, which has persuaded the Ministry of Health to give special focus on building a responsive and resilient health system in the second five-year health sector transformation plan (HSTP II 2021-2025). However, the years-long civil and armed conflicts have been fueling the COVID-19 crisis and have caused multi-sectoral infrastructure damage, human life loss, and economic crisis. In 2021 alone, the conflict causes more than five million internal displacements of persons (IDP) and thousands civilian deaths. Review of reported government data has shown that 3,508 health posts, 750 health centers, and 76 hospitals were partially or completely damaged in four regions. Looting of medical equipment and facilities for amenities was devastating. More than 19 million people were affected by the armed and civil conflicts between 2020 and 2021. Unless peace is ensured across the nation the sooner possible, it is foreseen that the devastation may further worsen, and recovery may be a far-fetched possibility. Therefore, in addition to restoration of the disrupted health services, it is the right time for the Ministry of Health to incorporate the humanitarian-development nexus as a joint strategy with the Disaster Prevention and Preparedness Commission (DPPC) to ensure a resilient health system for similar multifaceted conflict-related health crisis, disasters, and infectious outbreaks.

## Introduction

Ethiopia, the second most populous country in Africa, has made tremendous progress in the health status indicators over two decades (2000-2020), specifically achieving many of the millennium development goals, and was on track to achieve the sustainable development goals (SDG). However, the double burden (COVID-19 outbreak and years-long waves of civil conflicts in many areas of the country) has been challenging the health system. There was a growing concern that the longer civil conflicts last, the more likely the earlier all-round achievements get reversed (1).

For the earlier achievements, the government's commitment to universal health coverage (UHC) (starting from the time UHC was declared as one of the pillars of the SDG) (2) was unwavering. The 20-year Ethiopia's health envisioning (2015-2035) aspires to have a health status of a lower-middle-income country by 2025 and the median health outcomes of an upper-middle-income country by 2035, primarily by strengthening Primary Health Care (PHC) (3). The health sector transformation plan (HSTP I and II) and the essential health service package (EHSP 2019) underscore the move towards the achievement of UHC before the SDG due date (4,5). However, civil and armed conflicts are becoming among the major impediments to the progress.

Ethiopia has introduced the EHSP for essential PHC services to reduce financial hardship and upsurge health service utilization, thereby accelerating UHC (6). The progressively declining total fertility rate, growing contraceptive use, and expanding formal and informal education have been contributing to the observed demographic transition at national and regional levels. That is why earlier Ethiopia was recognized as a role model for raising the economy and health status among low-income countries (7). COVID-19 is ascribed for 6.8 million deaths globally, 258,000 (3.8%) and 7,500 (0.1%) deaths in Africa and Ethiopia, respectively (8).

While the country was struggling with civil conflicts and COVID-19 pandemic, a large-scale armed conflict was declared by the government of Ethiopia in early December 2020, following a provocative military action on the national defense force by the Tigray special force. After eight months of onset, the war spread to North Amhara and Afar regions and has cost the country a large number of human lives, infrastructure damage, and 28 billion USD losses as the Ministry of Finance publicly described.

The current assessment covers Amhara, Afar, Benishangul-Gumuz, Oromia, Tigray, and SNNP regions (Konso Zone). Regional and federal government damage assessment reports, emergency and conflict situation updates, program assessment reports, and development partner reports were used as source of data. The descriptions and statistics for Tigray region are based on the “Emergency Recovery plan published in June 2021” (9).

The purpose of this narrative analysis is to shed light on the health impact of the protracted conflicts and suggest policy directions to catch up with and ensure the achievements of the UHC and SDG health targets by making the health system resilient to natural and manmade shocks.

**Pre-armed-conflict health status indicators and financial inputs**: The 20-year National Health Sector Development Program (HSDP) (1990-2015) was not progressive but has served as a learning curve for the launching of HSTP I, which resulted in a remarkable and multidimensional health status improvement. Among others (Table 1), the remarkable improvement in maternal and child health, the highly diversified and near-universal vaccination coverage, the very successful malaria, TB, and HIV prevention and treatment program, the accelerated growth of the human resource for health development, the significant increment in PHC infrastructure constructions, and the huge investment for health facilities capacity building have encouraged the country at large to envisage achieving SDG targets (5,7,10-13). Nevertheless, these achievements may not qualify as being proxy and broad indicators for improvement in equity and quality of preventive and curative services at the subnational levels; between 1990 and 2019, substantial variations was noted in some of the indicators (12).

**Table 1:** The change in the national health status and health-related indicators over 30 years

Health indicators	1990	2020	Change (+/-)
**Health facilities**:			
Hospitals (hospital to population ratio)	96(1:500,000)	367(1:280,000)	3.8-fold
Hospital beds/10,000 population	3.2	2.7	1.2-fold
Health centers (health center to population ratio)	282(1:170,000)	3777(1:27,000)	13.4-fold
**Health workers**:			
Physicians (physician to population ratio)	1415(1:34,000)	12,314(1:8,000)	8.7-fold
Nurses (nurses to population ratio)	4774(1:10,000)	69,550(1:1,500)	14.6-fold
**Reproductive, maternal and child health**:			
Modern contraceptive utilization (married)	4.8%	41%	8.5-fold
Total fertility rate	7.3	3.8	1.9-fold
Antenatal care (at least one visit)	25%	74%	3-fold
Skilled person attended delivery	5%	50%	10-fold
Maternal mortality ratio/100,000 live births	1250	267	4.7-fold
Children fully vaccinated (<24 months age)	14%	44%	3.1-fold
Perinatal mortality rate/1000 births (2000 vs 2016)	52	33	1.6-fold
Neonatal mortality rate/1000 live births	50	27	1.9-fold
Under 5 mortality rate/1000 livebirths	204	55	3.7-fold
**Highly fatal communicable diseases**:			
Tuberculosis incidence/100,000 population	367	134	2.7-fold
Tuberculosis mortality rate/100,000 population	89	5	17.8-fold
HIV prevalence in % (1997 vs 2019)	3.4	0.9	3.8-fold
Malaria case fatality rate/100,000 population at risk	101	4	25.3-fold
**Other indicators**:			
Life expectancy	45	68	1.5-fold
Government expenditure on health as percentage of total budget	5.2	7.8	1.5-fold
Population in million	48	118	2.5-fold

Data sources: UN, Interagency working group (WHO, UNICEF, UNFPA, UNDESA, World Bank) estimate for 1990 to 2020, EDHS 2000-2019, MoH health and health related indicators

The number of hospitals at national level has increased by more than.8-fold, but the number of hospital beds per 10,000 population has reduced from 3.2 in 1990 to 2.7 in 2020, indicating that almost all new hospitals constructed are primary hospitals with a maximum of 42 beds for admission (5-7). Over the years, the contributions of development partners as bilateral, multilateral, and philanthropic donors for health status improvement have been immense. The share of donors for the total health expenditure in 2019/2020 was 34%, which was higher than the 30% average for low-income countries, but much lower than its share in 2010/2011 (50%). The government expenditure on health as a percentage of the gross domestic product (GDP) remains almost flat over a 20-year period. The government expenditure on health as a percentage of the total government expenditure has increased from 5% in 1995 to 8.5% in 2019/2020 (14), far from the Abuja Declaration target (15% of the annual budget), set in 2001 by the member states of the African Union (15). The modest increment was mainly as a result of the fiscal space of the progressively increasing government budget.

## The armed-conflict has fueled the civil-conflicts and COVID-19 pandemic weakened health system

**Health service delivery disruption and health infrastructure damage**: Ethiopia has been experiencing civil and armed-conflict breakouts since 2017, which have been ravaging the primary health care facilities, ambulances, and field vehicles. The health sector was also jeopardized by the indirect effect of the socioeconomic crisis of the conflicts, including damage to other sectors and private infrastructure, reduced agricultural activities, reduced productivity, crippled trade and commodity exchange, less interest to new investment, frequent disruption of primary to tertiary levels education, and roads blockage.

Between February and December 2020, it was a historic moment that health facilities providing curative services for non-emergency cases were nearly shut down due to COVID-19. Since both the health service providers and patients had been in extreme fear of acquiring COVID-19, a small number of critically ill patients and laboring mothers were visiting hospitals. The government was struggling to find personal protective and intensive care equipment, patient beds, and rooms. Assuming the worst to come, unfinished buildings and big hotels were prepared to serve as emergency hospitals, but when the time comes, that was not the case.

Later, while the health system was struggling to revive from COVID-19 wide-ranging negative effects, the armed conflicts erupted and damaged/looted health facilities (Table 3), including 76 Hospitals, 750 health centers, and 3,508 health posts (16). Basically, the armed conflict has fueled the COVID-19 pandemic and civil conflict-related disrupted health systems.

**Table 3:** Percentage of physically damaged health facilities out of the total available, by region*

Region	Health Facility Type	Partially damaged	Completely damaged	Total damaged	Percentage
**Afar**	Hospital	2	---	2/7	28.6
	Health Center	20	1	21/97	21.6
	Health Post	56	3	59/343	17.2
**Amhara**	Hospital	38	2	40/88	45.5
	Health Center	429	23	452/877	51.5
	Health Post	1642	86	1728/3565	48.5
**Benishangul-Gumuz**	Hospital	---	---	---	---
	Health Center	14	1	15/60	25.0
	Health Post	169	9	178/424	42.0
**Oromiya**	Hospital	---	---	---	---
	Health Center	142	7	149/1411	10.6
	Health Post	929	49	978/7099	13.8
**Tigray**	Hospital	32	2	34/41	82.9
	Health Center	107	6	113/226	50.0
	Health Post	537	28	565/743**	76.0

* The sources of data for the denominators are MoH health and health related indicators, 2019/2020.

** Extrapolated from the health centers damage.

**The effect of the conflict on civilians**: The conflict has brought devastation to human life both from direct physical harm and indirect effects of health system collapse. The current assessment has shown that out of nearly 71 million people in the aforementioned regional states and Konso zone, 19,252,959 (27%) people were affected by the conflict between 2020 and 2021. The proportion of the conflict-affected population was incomparably higher in Tigray and Afar, but the actual number of people affected in Amhara and Oromia each was nearly two-fold higher than the Tigray regional state (Table 4).

**Table 4:** The percentage of population affected by the conflict in the respective regions, 2020 -2021

Region/Zone	Total population in the region in million	Population affected by the conflict in million	Percentage
**Tigray**	5.6	4.5	80.4
**Afar**	2.0	1.4	70.0
**Amhara**	22.5	8.9	39.6
**Oromia**	39.1	8.6	22.0
**Benishangul Gumz**	1.2	0.4	30.6
**Konso zone**	0.4	0.08	20.0
**Total**	70.7	19. 0	27.0

The large-scale armed conflict continued for two years in the Northern, North Eastern, and Western regions, thereby resulting in millions of internal displacement of persons (IDPs), thousands human life losses, migration, and public infrastructure damages (16,17). According to the International Organization for Migration (IOM) Displacement Tracking Matrix 2021 report, Ethiopia was ranked top in the world for having unprecedented IDPs, virtually everywhere in the country (18). The contribution of the armed conflicts to the IDPs was exceptionally overwhelming, which is not an exception from the global experience, whereby 87% of IDPs at the end of 2020 were due to violence and armed conflicts (19).

Violations of international humanitarian law (IHL) and fundamental human rights of the civilian society were reported, and many were unaccountable to justice. IHL prohibited acts (“attacking civilians and civilians' property, starving of civilians as a method of warfare, committing sexual violence, reprisals, the destruction of objects essential to their survival, and the obstruction of relief supplies and assistance necessary for the survival of the civilian population”) (19) were reported by the Human Rights Watch and United Nations. The Ethiopian Human Rights Commission (EHRC) has also released a report on the violation of human rights (17).

Furthermore, American State Dpartment reported the prevailing sexual and gender-based violence (SGBV), including gang rape by military personnel in Tigray and Amhara regional states (21). Humanitarian crises were compounded by health risks associated with the interruption of regular preventive and curative health services. The physical and psychological traumas, and to the worst, the civilian human life losses were different from other conflict-affected countries (22).

The World Bank study in 2017 focusing on estimating the economic damage in the Syrian conflict concluded that “more people may have been killed due to a breakdown of the health system than due to direct fatalities from the fighting” (23). In the Amhara region alone, a rigorous unpublished study report has shown that nearly 7,000 civilians were killed during the armed conflicts. Amhara region has also estimated the infrastructure damage as costing more than five billion USD.

According to the IOM report, more than 5 million Ethiopians were identified as IDP in 2021 (18). The IDP reports from regional states (5.7 million) and UN estimates (5.6 million) (24) were comparable. The global estimate in 2019 has shown that more than 1.6 billion people (20%) were living in a humanitarian crisis, for which IDPs took quite a small share, 55 million (0.7%). Of these, 39 million (71%) were children and women (18,25). In 2021, 23.5 million people (53% children and 24% women) were on humanitarian assistance. Of these IDPs and IDP returnees accounted for more than 17% (26).

Studies from similar conflict-affected areas and other causes of the humanitarian crisis have shown that women, girls, and children were at increased risk of multiple problems, including SGBV and associated unplanned pregnancy and unsafe abortion, acquiring sexually transmitted infections (including HIV), exacerbated gender inequality, physical and long-lasting psychological trauma (27-30). Adolescent girls in IDP centers and armed conflict areas, in particular, were at increased risk of early sexual activity due to rape, and transactional sex as means of livelihood and safety (for socialization or protection from extra threats, including gang rape and homicide) (31,32).

In 2018, the UNFPA reported that over 500 women and girls daily die during pregnancy or delivery in crisis-affected areas, primarily due to a lack of access to optimal emergency obstetric care and abortion services (33). Unsafe abortion rate and maternal mortality had increased during an intensive phase of the conflicts; up to 61% maternal deaths globally occur in humanitarian crisis (34,35). There is also a large body of data that has shown the increased mortality of under-five children in IDP centers due to malnutrition, malaria, diarrheal and respiratory diseases, and other communicable diseases (31). Young people living in the conflict zone usually remain idle and hopeless, tempted to take risks, including the use/abuse of substances and participation in sexual and other human rights violence, creating a vicious cycle of the health crisis (36).

The suffering of older people from physical and psychological trauma and the worsening of chronic illnesses (due to lack of services) is another dark side of the conflict. While the breakdown in social norms and lawlessness exposes vulnerable people to physical and psychological trauma, adult men as well sustain physical injuries and are at increased risk of death and disabilities directly caused by armed conflict and physical violence (37).

**The public health consequences of the conflict**: Beyond internal displacement and mass exodus, the civil and armed conflicts in Ethiopia have hugely disrupted the regular preventive, promotive, and curative health services; and massively displaced health workers for more than a year in conflict affected areas, thereby increasing some communicable diseases outbreaks, and feared to cause a resurgence of eliminated diseases.

Unhygienic conditions, overcrowding, and malnutrition in IDP centers are major factors for the increased risk of outbreaks of communicable diseases (38-40). Mental health problems associated with the bad experience of the conflict, psychological stress, and substance use, therein persuaded by isolation, separation, inability to cope with new environment and people, interruption of education or employment are more prevalent among conflict-affected and displaced people (31,41).

As the Ethiopian large scale armed conflict continued for nearly two years, in the worst scenario, the health condition of the people in the conflict areas may not be that much different from the old times (in the 1800s), whereby the child mortality estimate was in the range of 43-51% (while the current global estimate is <5%) and maternal mortality ratio out of 100,000 live births was 1400 in Sub Saharan Africa (SSA) (42,43), while the recent estimates for SSA and Ethiopia were 545 and 267/100,000 live births, respectively (44).

The good work progress for the earlier achievements (such as 74% at least one antenatal care visit, 50% skilled persons attended delivery, 93% full vaccination, 41% modern contraceptive utilization) (Table 2) (45,46) was abruptly stopped during the conflict and COVID-19 outbreak, implying the increased disease burden and mortality therein, invariably reversing to the traditional way of life. Ethiopia has reduced tuberculosis incidence and mortality in two decades by 63% and 94% between 1990 and 2020, respectively, partly because the country had been relatively in stable condition, after nearly two decades of civil war (4).

**Table 2:** Pre-conflict baseline situation of some of the maternal and child health status indicators, 2019-2020

Indicator	National	Tigray	Amhara	Afar
Use of family planning method	41%	37%	50%	13%
Unmet need for family planning	22%	18%	33%	25%
Skilled birth attendance	50%	73%	55%	28%
Antenatal care 4+ visits	43%	61%	51%	46%
Caesarean delivery	5.4%	6.9%	7.4%	2.7%
Neonatal mortality rate/1000 live births (LB)	27	28	46	22
Under-five mortality rate/1000 LB	55	43	69	58
Fully vaccinated children <24 months	43%	73%	60%	20%

Data sources: EDHS 2016 and 2019, MoH health and health related indicators

Pragmatically, people in the conflict areas have been suffering from a double-edged sword (the direct effect of the war and lack of basic health services and humanitarian support); sadly, the majorities are poor, disabled, and aged. Their suffering due to the aftermath of the armed conflict is usually protracted and may be more catastrophic than the conflict when access to humanitarian and emergency health service needs is suboptimal (40,47).

The returnees after protracted displacement as well suffer from psychological stress associated with frustration of resumption of conflict, going to ghost villages, and damaged personal and public properties. Some may dispossess their lands, houses, pet animals, and other personal properties (48). As learned from other countries, returnees to urban areas were disproportionately unemployed and poorer than the general population (49).

**The health system response to the crises**: While the crises were mounting, the Ministry of Health (MoH) developed a health recovery and restoration plan to enhance rapid recovery of the healthcare system in conflict affected areas. The focus was restoration of health infrastructure, health information system, essential health services, and pharmaceutical supplies. The health crisis as well demanded the establishment of National and Regional Public Health Emergency Operations Centers (PHEOC) (50).

Since the majority of the health facilities in armed conflict areas were damaged, Mobile Health and Nutrition Teams (MHNT) and temporary IDP clinics were established and deployed to provide the basic services. Since the SGBV cases were rising, 16 centers (9 one stop centers and 7 Integrated GBV centers) were established to treat the survivors. In addition, 2000 volunteer health workers were deployed to more than 200 health facilities.

**Ensuring a resilient health system: Building the humanitarian-development nexus**: Unfortunately, Ethiopia has been experiencing civil and armed conflicts covering larger areas and resulting in formidable multi-sectoral destructions and human life losses. The health system has been one of the most affected public sectors. As a short-term plan, speeding up the conflict resolution and ensuring sustainable peace is not arguable to resume the health services and bring about cohesion with the other sectors and the people. As a long-term preparation and response, the health sector should have system-based coordination and preparation with other sectors and partners to minimize the devastating effect of unavoidable disasters and ensure a resilient health system in a state of fragile condition.

The commonly observed emergency response in crisis-affected areas is humanitarian aid and emergency health care; the developmental health services are usually given little attention if not unavailable. The currently pressing issue is that, unless the post-conflict-crisis management is coordinated by intertwining humanitarian aid and developmental interventions to reach the IDPs and returnees, business as usual delays the recovery and rehabilitation process and interrupts the regular preventive, promotive, and curative health services.

It was also noted that economic, security, and psychosocial factors are major determinants of the IDPs' preference to return home (51). The UN has noticed the development intervention gap in the conflict and natural disaster-related crisis areas since 2016 and recommended establishing humanitarian-development-peace nexus (HDPN) as a strategy to make the health system resilient, avoid further damage, and sustain peacebuilding (52). Following this recommendation, the UNFPA has taken part by providing life-saving sexual and reproductive health services in times of crisis in over 55 countries (53).

**Figure 1:**
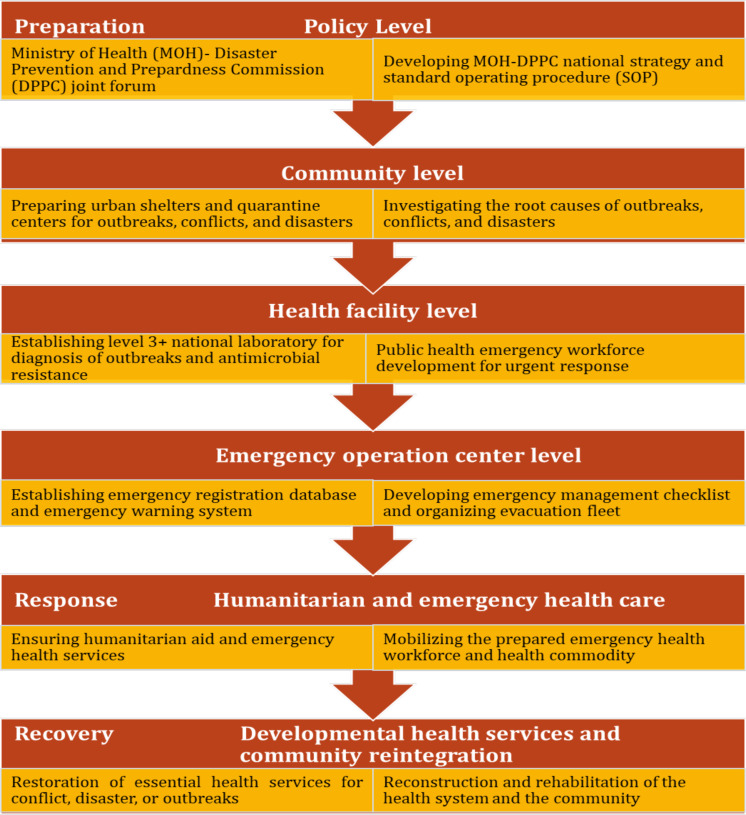
The Humanitarian-Development nexus framework for preparation, response, and recovery

HDPN is an innovative approach for the health system to optimally manage the health crisis due to conflict or disaster and ensures the continuity of the developmental health services during the post-conflict period. Basically, it is all about bringing humanitarian and development agents to plan together, get prepared, and create an interface in the emergency and recovery responses. The jointly planned preparation, response, and recovery interventions are summarized in Figure 1. It is underscored that joint humanitarian and development actions become effective when peace is ensured.

In the Ethiopian situation, as humanitarian aid is led by the DPPC, bringing MoH and DPPC to form a joint body is the first step towards the realization of the HDPN. The joint action enables developing a joint policy, setting up a joint steering committee overseeing humanitarian support, emergency health recovery, a continuation of the development of health services, and rehabilitation process in the conflict or disaster-affected areas. As such undertaking is in the interest of the UN and its agencies, it avoids discursive efforts and enables better resource mobilization and utilization (54).

Beyond DPPC and MoH synergistic actions, the HDPN requires multiparty coordination and alignment. As the two wings have different donors, and have a unique institutional scope and mandate, a careful orchestration of donors and implementing partners is critically important to set up a seamless interface and have a better outcome for the HDPN. Otherwise, attempting to create cohesion between the humanitarian (which follows the principle of neutrality, impartiality, and independence) and development (whose approach is long-term, political, and rights-based) may take a very long time if not impossible (54). In other words, an exogenous and international action (humanitarian) may not soon interface with the Ethiopian government's interest (development) unless a joint consensus is built with discussion for action. The coordination role played by the Ministry of Finance, a continuation of the earlier established National Disaster and Risk Management Committee platform, to bring stakeholders together for a common goal and better mobilize resources can be taken as a learning curve.

The great advantage of interfacing the development with humanitarian actions is to better mobilize resources and ensure the effectiveness of recovery and rehabilitation of the conflict/disaster-affected population by aligning their policies and approaches (54,55). In the emergency response, in particular, the integration of development health services into the humanitarian crisis response has got increasingly global focus (56). Therefore, realizing the HDPN is for better preparation and timely coordinated action in humanitarian and fragile settings.

Further, the establishment of a resilient health system for any shock will be better resourced and coordinated if a health strategy in humanitarian settings is implemented. The emergency preparedness and response guide for sexual and reproductive health in humanitarian settings could not address other developmental health activities, including child health, nutrition interventions, vaccination, communicable diseases prevention and treatment, water, sanitation and hygiene, neglected tropical diseases prevention, global health focus areas (HIV, malaria and tuberculosis) prevention and treatment amongst others.
